# In Vitro Evaluation of Tooth-Whitening Potential of Peroxide-Free OTC Dental Bleaching Agents

**DOI:** 10.3390/dj11040089

**Published:** 2023-03-27

**Authors:** Marlene Grillon, Enrico Di Bella, Ivo Krejci, Stefano Ardu

**Affiliations:** 1 Division of Cariology and Endodontology, Section of Dental Medicine, Faculty of Medicine, University of Geneva, 1205 Geneva, Switzerland; 2 Department of Political and International Studies, University of Genoa, 16126 Genoa, Italy

**Keywords:** ∆E00, peroxide-free dental bleaching, over-the-counter, spectrophotometer

## Abstract

**Purpose**: To evaluate and compare the tooth-whitening potential of five over-the-counter (OTC), peroxide-free dental bleaching methods as well as an experimental tooth-whitening solution containing 0.1% hydrogen peroxide complexed with doping agents with a gold standard (positive control) containing 16% carbamide peroxide. **Material and Methods**: Eighty permanent bovine incisor teeth were randomly allocated to eight different groups. Two teeth from each group were immerged into five staining solutions represented by coffee, tea, red wine, and curry mixed in warm oil or distilled water (control group) and stored at 37 °C for 28 days in an incubator. The teeth were then reallocated to the eight groups, resulting in ten samples per group, and each group was matched with a bleaching product. The bleaching procedures were executed following the manufacturer’s recommendations. The color of each sample was assessed over a white and black background using a quantitative numerical measurement approach with a calibrated spectrophotometer. Spectrophotometric measurements were performed after exposing the teeth to the bleaching agent for 60 min (T2), 100 min (T3), and 200 min (T4), and ΔE00 was calculated. **Results:** When analyzed over a white background, the mean ΔE00 values ranged from 2.14 (placebo) to 6.32 (Opalescence PF). When analyzed over a black background, the mean ΔE00 values ranged from 2.31 (placebo) to 5.78 (Opalescence PF). Statistically significant ΔE00 color changes over time for the eight groups and five staining solutions at T1 and T4 were assessed for both backgrounds using repeated ANOVA followed by Fisher’s LSD post hoc test (*p*-value < 0.01). **Conclusions:** All tested over-the-counter whitening kits except one exhibited positive color variation. However, the individual performance differed vastly from one brand to the other, and the overall performance was less effective compared to the conventional carbamide-peroxide-based positive control.

## 1. Introduction

Aesthetic dentistry has grown in popularity, and the appearance of teeth is an increasingly important priority among patients and often associated with health and beauty [1,2]. As the world population is aging more than ever, more resources are allocated to the quest for eternal youth to fight against the negative effects that aging has on the physical appearance [3]. Aesthetic dentistry is not immune to this trend. On the contrary, the smile takes a central role in overall beauty, and smile makeovers are highly demanded by the population. When it comes to dental beauty, there are many factors that can affect the perfect smile: tooth decay as well as pathologies such as traumas or orthodontics complications might have a high impact on the form and shape of the smile. Finally, abnormal teeth coloration or translucency might cause distress and affect the overall appearance of the smile. The key staining causes are typically classified into two main categories: extrinsic causes and intrinsic causes [4]. The extrinsic causes are the ones driven by the behaviors of the individual, such as particular dietary choices. On the other hand, the intrinsic factors are completely unrelated to specific behaviors but driven by different health pathologies occurring before or after birth, such as fluorosis, tetracycline stains, and others. Patients unsatisfied with their teeth color appearance may resort to a tooth vital bleaching [5]. According to the American Academy of Cosmetic Dentistry, tooth vital bleaching is the most commonly requested treatment in dental offices across the Unites States of America [6]. Diagnosis plays a crucial role in proposing the right approach, and depending on the staining cause, the treatment might be different. Historically dental bleaching is performed in dentals offices or at home under supervision. Starting from 1990, a new form of dental bleaching emerged and is sold over the counter (OTC) [7,8]. In recent times, OTC products, which are more affordable than traditional dental office bleaching, have proven to be very successful, especially among young adults [9]. Despite being freely available on the market (OTC solution can be used at home without the supervision of a professional), these products are not risk-free and might generate side effects such as hypersensitivity and gingival irritations [10]. In 2012, the European council enacted a new law concerning cosmetic products and also targeting dental bleaching solutions containing hydrogen peroxide [11]. Today, the number of research papers analyzing the efficiency and side effects of these products is not exhaustive enough to draw a firm conclusion regarding their effectiveness. The objective of the present study is to evaluate the tooth-whitening potential of five OTC peroxide-free dental bleaching methods as well as an experimental tooth-whitening solution containing 0.1% hydrogen peroxide complexed with doping agents. These results were compared with those of the gold standard (positive control) containing 16% carbamide peroxide.

## 2. Materials and Methods

### 2.1. Sample Selection and Preparation

Eighty permanent bovine incisors were extracted, stored in water, and randomly allocated to eight different groups with ten teeth each. The roots were sectioned 1 mm below the cementoenamel junction (CEJ) using a slow-speed water-cooled diamond saw (Minitom, Struers Type 04436216, Serial No. 44310284). All teeth were carefully cleaned with pumice and numbered in the palatal area with a bur. All operations were executed by the same operator. Sample selection and preparation were performed following the methodology described by Dietschi et al. [12].

### 2.2. Staining Procedure

Five different staining solutions were readied for this study and kept in plastic bottles (Table 1): group A: 60 mL coffee (Ristretto, Nespresso, Nestle, Lausanne, Switzerland); group B: three tea bags in 60 mL of boiling water (Twinings Earl Gray tea, London, UK); group C: 60 mL red wine (Côte du Rhône (DOC), Les arénes, Vacqueyras, France); group D: 5 g of curry mixed in 60 mL of warm oil (Curry Bio Natura plan Coop, Switzerland); group E: 60 mL distilled water (control group). Two teeth from each group were immerged into the respective staining colorant and stored at 37 °C for 28 days in an incubator (INP-500, Memmert GmbH & Co.KG, Schwabach, Germany). The staining solutions were renewed every 7 days to avoid bacteria or yeast contamination. After 28 days of storage, the teeth surfaces were cleaned using a high-pressure hot-water airbrush (0.4 MPa 135 °C, Minivapor 93, Effegi Brega s.r.l., Sarmato, PC, Italy) and briefly air-dried. All details regarding the staining methodology were described in previous publications [13,14].

### 2.3. Bleaching Procedure

After the staining procedure, the stained teeth were reallocated to the eight groups, resulting in ten samples per group, and each group was matched with a bleaching product (Table 2): group 1: MeaWhite kit teeth whitening (MEA); group 2: iWhite instant teeth whitening (IWH); group 3: PAP pure (PAP pure); group 4: Opalescence PF 16% (OPL); group 5: Experimental bleaching agent (EXP1); group 6: Hismileteeth (HST); group 7: placebo (GLY); group 8: oZoral gel oral (OZG). The bleaching procedure consisted of thoroughly covering the entire surface of the enamel with the bleaching agent to ensure even distribution of the product. A thickness gauge was used to measure the thickness of the layer, which was approximately 1 mm thick. The agent was left on the surface of the enamel for the defined period of bleaching, then rinsed with water for 30 s, and the surface was then cleaned with paper tissue. All applications were performed following the manufacturers’ recommendations (Table 3). For each group, the bleaching gel was applied for 60, 100, and 200 consecutive minutes. During the bleaching periods, samples were kept at ambient temperature and 100% humidity. Following the manufacturer’s recommendations, the applications of MEA/IWH/PAP/OPL/GLY/OZG were repeated every 20 min, EXP1 every 60–100–200 min, and HST every 10 min. Moreover, the bleaching procedures for MEA, PAP, and HST were always combined with light activation (Bluephase G2, IvoclarVivadent, Schaan, Lichtenstein) (standardized distance of 2 mm), in line with the manufacturer’s recommendations.

### 2.4. Color Change Measurements and Data Collection

The color of each sample was recorded on a black and on a white background using a quantitative numerical measurement approach with a calibrated spectrophotometer (Spectro-Shade, Handy Dental Type 713000, Serial No. HDL0090 MHT). The classic CIEDE 2000 (ΔE00) formula based on lightness (ΔEL), chroma (ΔEC), and hue (ΔEH) was used to determinate color changes [14,15]. Spectrophotometric measurements were performed after exposing the teeth to the bleaching agent for 60 min (T_2_), 100 min (T_3_), and 200 min (T_4_), respectively. Before every spectrophotometric measurement, the samples were stored in distilled water at room temperature for 24 h to avoid dehydration. An integrated detection function within the spectrophotometer guaranteed equal measurement conditions for all measurements due to reproducible positioning perpendicular to the sample surface. Before every measurement, the spectrophotometer was calibrated using the green and white calibration standard provided by the manufacturer. A D65 (6500 °K) light source illuminating simultaneously from both sides at a 45° angle was used for the measurements, and the system’s detector area received a 0° angle reflected light. Data generated from the spectrophotometer were stored in a proprietary image file format [14]. For each tooth image file, six measurements were taken on different zones based on a clockwise sequential localization in order to generate details of CIE L*a*b data. CIE L*a*b values were recorded at the beginning of the study on the unstained extracted teeth (T0). Another measurement was taken after the staining procedures in order to evaluate the staining susceptibility (T1). Finally, measurements were taken after each bleaching step (T2, T3, and T4). Based on the L*a*b scores, color changes were calculated using the classical CIEDE 2000 (DE00) formula [16,17].

## 3. Statistical Analysis

Statistically significant CIEDE 2000 color changes over time for the eight groups and five staining solutions were assessed using repeated ANOVA measures with sigma restricted parametrization to account for categorical predictors in the model, followed by Fisher’s LSD test (*p*-value < 0.01). Samples ranked with the same letter were considered equivalent in terms of color change. Normality assumptions were checked using the Shapiro–Wilk normality test on the within-cells residuals of the ANOVA analysis (*p*-value > 0.1). All statistical analyses were performed in Statistica 13 (Tibco Software Inc., Palo Alto, CA, USA). CIEDE00 color differences were computed in MATLAB 2017b (The Mathworks, Inc., Natick, MA, USA)

## 4. Results

**Figure 1:**
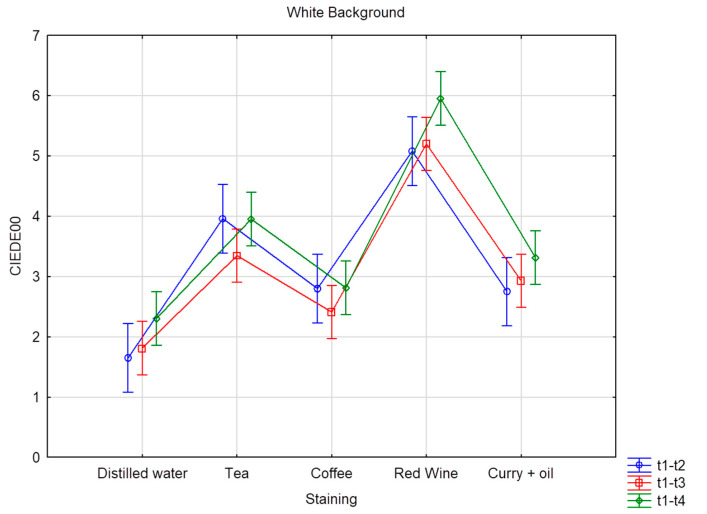
Average color difference in terms of CIEDE00 over time according to different staining liquids analyzed over a white background. Vertical bars denote 95% confidence intervals.

**Figure 2:**
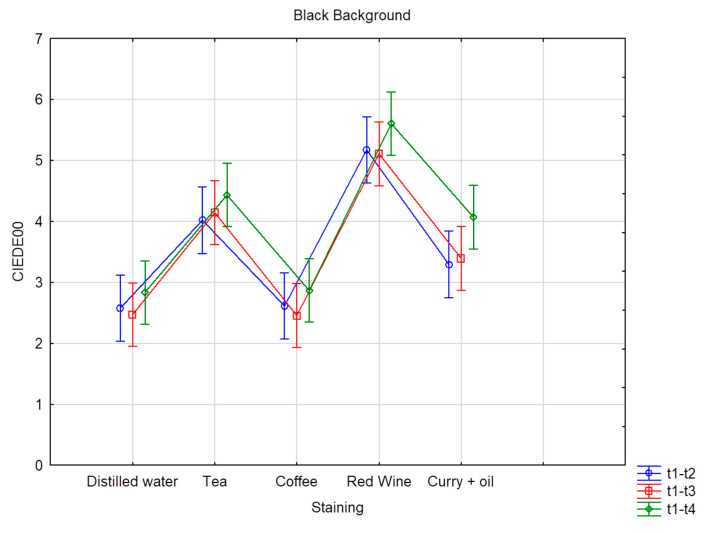
Average color difference in terms of CIEDE00 over time according to different staining liquids analyzed over a black background. Vertical bars denote 95% confidence intervals.

**Figure 3:**
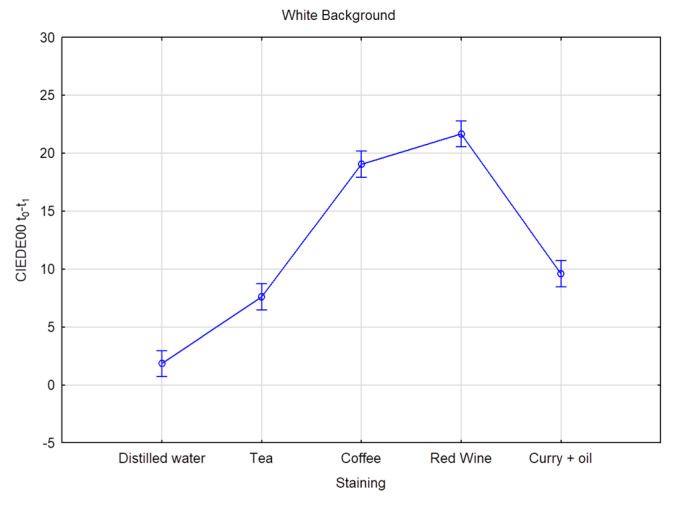
Average color difference in terms of CIEDE00 (t0-t1) according to different staining liquids analyzed over a white background. Vertical bars denote 95% confidence intervals.

**Figure 4:**
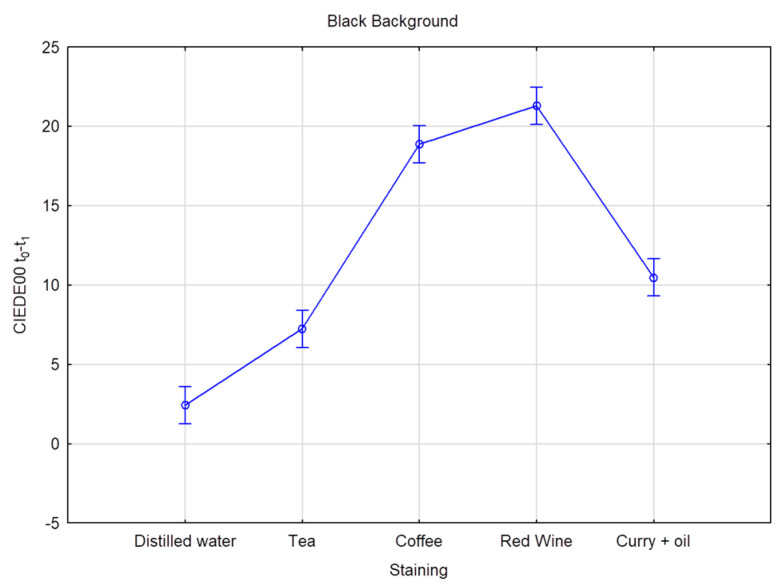
Average color difference in terms of CIEDE00 (t0-t1) according to different staining liquids analyzed over a black background. Vertical bars denote 95% confidence intervals.

**Figure 5:**
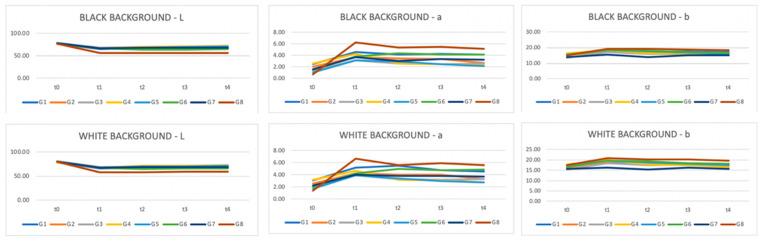
L*a*b* change over time for every bleaching agent over a black and a white background.

Six CIE L*a*b measurements were recorded on each of the 80 teeth, resulting in 480 measurements per time interval, totaling 2400 measurements for the five times intervals. Table 4 and Table 5 provide the mean and standard deviation CIEDE00 color changes over time for all the groups and staining solution, on both the black and the white background, respectively. The overall color change considers the data pooled together per bleaching product but without distinction per staining liquid. Superscripts denote the samples’ ranking for each staining solution and time. Superscript A corresponds to the best and D to the worst ranking. Results with the same superscript are not significantly different according to Fisher’s LSD test; *p* value < 0.01. The highest DE00 value represents the highest color change difference. On the white background, when stained by distilled water, values ranged from DE00 3.26 (Opalescence PF) to 1.04 (oZoral Gel), with no significant differences between the bleaching products. On the white background, when stained with coffee, bleaching susceptibility values ranged from DE00 3.69 (EXP1) to 1.54 (Glycerin), with no meaningful statistical differences observed. On the white background, when stained with curry mixed with oil, bleaching values ranged from DE00 5.07 (EXP1) to 2.13 (PAP pure), with significant differences observed. On the white background, when stained with red wine, bleaching values ranged from DE00 11.2 (Opalescence PF) to 2.86 (oZoral Gel), with significant differences being present. On the white background, when stained with tea, bleaching values ranged from DE00 10.17 (Opalescence PF) to 2.09 (Glycerin), and here again, significant differences were observed. The overall color change on white background ranged from DE00 6.32 (Opalescence PF) to 2.14 (Glycerin), with significant differences between the products. On the black background, when stained with distilled water, bleaching values ranged from DE00 4.83 (Opalescence PF) to 1.25 (oZoral Gel), with significant differences. When stained by coffee and measured on the black background, bleaching values ranged from DE00 4 (iWhite) to 1.73 (Glycerin), without being significantly different form each other. On the black background, when stained by curry mixed with oil, bleaching values ranged from DE00 6.02 (EXP1) to 2.36 (PAP pure), and the differences were statistically significant. When stained with red wine, bleaching values ranged from DE00 9.39 (Opalescence PF) to 3.03 (Glycerin) on the black background; the differences were also statistically significant. When stained with tea, bleaching values on the black background ranged from DE00 7.73 (Opalescence PF) to 1.77 (Glycerin), and the differences were statistically significant. The overall color change measured on black background was significantly different between the bleaching products and ranged from DE00 5.78 (Opalescence PF) to 2.31 (Glycerin) (Table 6 and Table 7), and Figure 1 and Figure 2 provide the mean and standard deviation CIEDE00 of the color difference over time among different staining liquids on the white and the black background, respectively. The total value color change considers the data pooled together over time without distinction per staining liquid. On a white background, the mean ranged from DE00 5.96 (red wine) to 2.30 (distilled water), with statistically significant differences observed and a total value of DE00 3.67. On a black background, the mean ranged from DE00 5.61 (red wine) to 2.84 (distilled water), with statistically significant differences observed and a total value of DE00 3.96. Table 8 and Figure 3 and Figure 4 provide the mean and standard deviation CIEDE00 of the color difference over initial time among different staining liquids on a white and a black background, respectively. On a white background, the mean ranged from DE00 21.67 (red wine) to 1.85 (distilled water), with significant differences observed. On a black background, the mean ranged from DE00 20.30 (red wine) to 2.42 (distilled water), with significant differences observed. Initial and final L*a*b values of the samples are illustrated in Table 9 and Figure 5.

## 5. Discussion

Considering the results of this study, OPL showed the highest ∆E00, thus having the best bleaching capacity. This bleaching agent was used as the positive control. Its high performance was thus expected and may be explained by its content of 16% carbamide peroxide. The good efficiency and bleaching effect of product composed of 16% carbamide peroxide was previously demonstrated [12].

EXP1 also showed high bleaching performance, with the second-highest ∆E00 value in this study. EXP1 is an experimental solution in which the active ingredient is composed of a low concentration of hydrogen peroxide (0.1%) mixed with a doping agent. More details about the exact composition of this new solution cannot be revealed at this time, as the patenting process is currently underway. Given the promising outcomes with such a low concentration of hydrogen peroxide, we may speculate that the aim of the doping agents is to boost the oxidation-reduction reaction. Further research on EXP1 will be necessary at a later stage to obtain more information on the product efficacity.

HST showed good results in terms of the absolute numbers. However, if we take the detailed L*a*b* values (Figure 5 and Table 9), we can conclude that the ∆L* and ∆b* did not change favorably with stains from tea and red wine. As mentioned previously, after an effective bleaching procedure, we expect an increase in the luminosity (∆L*) and a decrease of the yellow tone (∆b*). However, for those two cases, after the application of the bleaching agent, the ∆L* values went down, which represents a decrease in brightness, and ∆b* increased, which represents an increase in the yellowness. When it comes to stains from coffee and curry, ∆L* and ∆b* were positively impacted by the bleaching procedure. One assumption to explain these results may be the relationship between the chemical affinity and molecular polarity, suggesting that HST has a low affinity to staining agents with high polarity [13], as coffee has low polarity, while tea and red wine have high polarity [18,19,20]. HST not only showed a lack of whitening effect on the high-polarity yellow-staining agent and on red wine but even had a negative effect considering that teeth of these two groups appeared yellower and less bright after the bleaching procedure. Greenwall-Cohen and colleagues raised a public health concern regarding OTC whitening products presenting a lack of effectiveness. Due to the lack of efficiency, consumers will tend to overuse them with the aim of obtaining a favorable outcome. This trend has been described as a “catch up mentality” [21]. HST is composed of phthalimidoperoxycaproic acid (PAP) as the main active bleaching ingredient. Unlike hydrogen peroxide, PAP has another method of oxidation action that does not come from the oxidation-reduction reaction but comes from an epoxidation reaction, which as a result will form an epoxide (oxirane) product [22]. The concentration of every active ingredient has to be considered when analyzing the effectiveness of a bleaching solution; however, HST manufacturers do not reveal any information regarding phthalimidoperoxycaproic acid (PAP) concentration. Without further knowledge, we can hypothesize that the HST’s poor bleaching efficiency may be linked to a sub-optimal concentration of the active agent. Phthalimidoperoxycaproic acid (PAP) has been widely used among several industries besides dental bleaching. It is used as a bleaching agent for textiles, in cleaning and laundry products, as well as in personal care cosmetics including make-up, fragrance, and shampoo. Surprisingly, phthalimidoperoxycaproic acid (PAP) is also used in the agricultural sector, including as an active agent for pesticides [23].

HST displayed overall poor results and even worsened the appearance of teeth stained with tea and red wine. To better understand these results, we need to further investigate into HST composition. For example, Punica granatum seed (pomegranate) extract is one of its components, and the manufacturer declares the use of this ingredient is for its anti-inflammatory proprieties. Indeed, in the literature, pomegranate waste extract have been described for its ability to “scavenger free radical and its potent antioxidant capacity” as well as its “antibacterial, antiviral, hypolipidemic and anti-inflammatory” abilities [24]. In addition to these properties, another vein of research studied the “staining effect of pomegranate flower extract on human blood cells” and highlighted pomegranate flower extract’s staining capacity [25]. It is described as a “deep orange-brown neutral dye”, as pomegranate flower extract is able to stain human blood cells (which are pH-neutral). One assumption to explain the unfavorable results obtained when HST is used with teeth stained with red wine and tea may be related to pomegranate extract’s staining capability on pH-neutral substrates. Malir et al. showed that black tea beverages range around pH 6.68, which may be consistent with the “neutral dye” pomegranate staining ability [26]. When it comes to red wine, clear data about the pH are not available; however, the assumption is that it is acidic, and its pH range is below the neutral pH. Moreover, pomegranate’s deep orange-brown staining might explain the decrease in brightness (∆L*) observed with tea and red wine subtract. The increase of the yellowness (∆b*) could be explained by the Chamomilla recutita flower (chamomile) extract, which is also part of HST’s composition. According to the manufacturer’s description, this ingredient is used as a soothing agent and also for its anti-inflammatory properties. Chamomilla recutita flower (chamomile) extract is composed of a chemical compound called apigenin, which is part of the flavone class. Apigenin has a solid yellow, crystalline appearance and is known for its anti-inflammatory, antioxidant, and other properties. Moreover, due to its yellow appearance, apigenin has been used to dye wool [27]. Even though the HST manufacturer does not reveal the concentration of Punica granatum seed (pomegranate) extract and Chamomilla recutita flower (chamomile) extract, it is reasonable to assume that these two components play a role in the ∆L* and ∆b* variations. However, more research is needed to better explain this phenomenon.

MEA and IWH showed overall similar behavior. Both of them contain citric acid as an active agent, and additionally, IWH contains phthalimidoperoxycaproic acid (PAP). Citric acid is mainly found in fruit drinks or juices and is known for its erosive action [28]. The citric acid contained in these bleaching agents’ main action results in etching the tooth surface. It has a favorable action only with the pigments located in the external layer of the tooth rather than removing staining in the deep surface. In some studies, citric acid is also described as an accelerator for bleaching [29]. When it comes to IWH, the manufacturer does not reveal any details regarding phthalimidoperoxycaproic acid (PAP) concentration, which limits deep analysis. In addition to the previous active agent, hydrated silica is also present in IWH’s composition. Hydrated silica are abrasive particles that remove extrinsic stains by superficial abrasion and therefore result in a lightening effect [30]. It is mainly found in whitening toothpaste, and Mosquim et al. widely described its action and highlighted that these particles “enhanced the enamel erosive wear [31].

Phthalimidoperoxycaproic acid (PAP) is a non-hydrogen-peroxide active agent increasingly used in OTC bleaching agents. In order to assess and compare its whitening potential, this study selected three bleaching products, namely IWH, HST, and pure PAP, all containing this active agent. Each product displayed different outcomes. Pure PAP, with a concentration of 10–15% of the active agent, showed the lowest whitening potential in this study. Two assumptions can be made to explain these discrepancies: one related to the different concentration of the active ingredient present in each product and a second one related to the variations in the other ingredients constituting each product. Indeed, in addition to PAP as the main active ingredient, MEA and IWH also contain abrasive agents such as citric acid, hydrated silica, and sodium bicarbonate. The conclusion based on these observations is that PAP combined with an abrasive agent presents a more favorable overall bleaching outcome.

Finally, OZG demonstrated a very low whitening potential similar to the negative control (GLY). Ozonized sunflower seed oil is OZG’s main active agent. Due to its various biological properties, such as antimicrobial effects (bactericidal action), angiogenesis stimulation, and high oxidative capacity, ozone is considered a promising molecule [32,33]. Ozone has been used widely and successfully in dentistry. It is an unstable and very reactive gas with a short half-life, and for this particular reason, it cannot be stored [34]. Elements such as air, water, pH, and temperature will have an impact on its decomposition. To explain OZG’s poor whitening effectiveness, it can be assumed that ozone does not display a favorable result when used in the form of paste due to the presence of oxygen. The oxidative potential depends on ozone concentration; however, when it comes to OZG, the manufacturer does not provide any information in this regard. It can be assumed that ozone’s concentration and the radical’s formation may be insufficient in OZG. Lastly, the short contact time between the tooth and OZG paste might be unfavorable for deep action of the oxidative agent.

According to Table 8 and Table 9 and Figure 1 and Figure 2, the bleaching exposure time has a positive impact on the final color variation (∆E00). Moreover, when exposed to a bleaching agent, red wine represents the staining substrate providing the highest color variation (∆E00) over time.

## 6. Conclusions

The comparison between commonly used over-the-counter whitening kits and “traditional” products based on hydrogen peroxide resulted in three key takeaways:

1. All over-the-counter whitening kits tested in this study, except one, exhibited positive color variation. However, the individual performance differed vastly from one brand to the other, and the overall performance was less effective compared to the conventional carbamide-peroxide-based positive control;
2. One product, Hismileteeth, showed a partially negative performance with two specific staining agents. Further research might be needed to understand and investigate the disparity in performance driven by the underlying staining agent;
3. The experimental bleaching agent showed the best results of all OCT products tested. These results were close to the positive control with carbamide peroxide.

## 7. Limitations

This study was conducted in vitro, and while in vitro studies can provide valuable insights into the effects of different treatments on biological tissues, they do not fully capture the complexities of the oral environment in vivo. In particular, the presence of saliva in the mouth can influence the parameters we measured and the outcomes of our study.

## Figures and Tables

**Table 1:** Details of staining solution.

Group	Staining Agent	Manufacturer	Batch Number	Proportion
Group A	Coffee	Ristretto, Nespresso, Nestlé, Switzerland	0272378606	60 mL
Group B	Tea	Twining Earl Gray tea, London, England	0000579251	3 tea bags in 60 mL of water
Group C	Red Wine	Côte du Rhône (DOC), Les arènes, Vacqueyras	1306471D	60 mL
Group D	Curry	Curry Bio Natura plan Coop	1291177	5 g curry in 60 mL water
Group E	Distilled Water	N/A	N/A	60 mL

**Table 2:** Details of bleaching agents.

Group	Product	Manufacturer	Ingredients	Active Agent
**N°1**	MeaWhite kit teeth whitening	Plastimea SA (Brussel, Belgium)	Glycerin, Propylene glycol, Purified water, Hazel extract, Sodium phytate, Citric acid, carboxymethyl	Citric Acid
**N°2**	iWhite instant teeth whitening	Sylphar NV (Deurle, Belgium)	Aqua, Hydrated Silica, Glycerin, Sorbitol, Chondrus Crispus Powder, PEG-40 Hydrogenated Castor Oil, Aroma, Phthalimidoperoxycaproic Acid, Citric Acid, Methylparaben, Acrylates/Acrylamide Copolymer, Paraffinum Liquidum, Xylitol, Calcium Lactate, Calcium Gluconate, Potassium Acesulfame, Polysorbate 85, BHT	Phthalimidoperoxycaproic Acid, Citric Acid
**N°3**	PAP pure	Cosmolab (Zurich, Switzerland)	Glycerin, propylene glycol, maltodextrin, phthalimidoperoxycaproic acid, acrylates/C10–30 alkyl acrylate cross polymer, menthe arvensps leaf oil, mica, CI 77891, menthe piperita oil sodium saccharin	Phthalimidoperoxycaproic Acid (10–15%)
**N°4**	Opalescence PF 16% regular	Ultradent (Dardilly, France)	Carbamide peroxide 16%, Glycerin, Water, Urea, Xylitol, Carbomer, PEG-6, Sodium Hydroxide, EDTA, Potassium Nitrate, Sodium Fluoride	Carbamide Peroxyde 16%
**N°5**	EXP1	CUMD (Geneva, Switzerland)	0.1% H_2_O_2_, Doping agent	0.1% H_2_O_2_
**N°6**	HiSmile teeth whitening kit	HiSmile Pty Ltd (Goldcoast, Australia)	Sorbitol, Water, Phthalimidoperoxycaproic Acid, Propylene Glycol, Glycerin, Potassium Nitrate, Polyethylene Glycol-8, Hydroxyapatite, Sodium Carboxymethyl Cellulose, Hydroxyethyl Cellulose, Xanthan Gum, Peppermint Essence, Saccharin Sodium, Methylparaben, Sodium Bicarbonate, Aloe Leaf Extract, Chamomile Extract, Pomegranate Seed Extract, Propylparaben	Phthalimidoperoxycaproic Acid
**N°7**	Lubricating Gel	K-Y Johnson & Johnson	Water, Glycerine, Propylene Glycol, Hydroxyethylcellulose, Methylparaben, Sodium phosphate, Disodium phosphate, Propylparaben, Tetrasodium EDTA	N/A
**N°8**	oZoral Gel oral	Innovares Srl (Sant’Ilario d’Enza, Italy)	Water, Ozonized Sunflower Seed Oil, Aroma, Glycerin, Carbomer, Polycarbophil, Sodium Hydroxide, Sodium Saccharin, Glyceryl Caprylate, Tocopherol, Ascorbyl Palmitate, Disodium EDTA, Limonene, Linalool	Ozonized Sunflower Seed Oil

**Table 3:** Bleaching agent.

Group	Product	Code	Batch Number	Instruction for Use	Experimental Application	Light Activation
**N°1**	MeaWhite kit teeth whitening	MEA	93/42/EEC2007/47/EC	20 × 20 min	20 × 20 min	Yes
**N°2**	iWhite instant teeth whitening	IWH	AAA15605-2020	5 × 20 min	10 × 20 min	No
**N°3**	PAP pure	PAP pure	No batch number as is has been freshly produced in the manufacturer’s laboratory	10 × 20 min	10 × 20 min	Yes
**N°4**	Opalescence PF 16% regular	OPL	BGX34	7 × 5 h	10 × 20 min	No
**N°5**	EXP1	EXP1	No batch number as is has been freshly produced in the CUMD laboratory	10 × 20 min	10 × 20 min	No
**N°6**	HiSmile teeth whitening kit	HST	111042019	6 × 10 min	20 × 10 min	Yes
**N°7**	Gylcerin	GLY	8351914	N/A	10 × 20 min	No
**N°8**	oZoral Gel oral	OZG	30318	7 × 20 min	10 × 20 min	No

**Table 4:** Average CIEDE00 color changes over time and standard deviations (in parentheses) per group analyzed over a white background and corresponding grouping (A, best; D, worst). Results with the same capital letter are not significantly different according to Fisher’s LSD test; *p* value < 0.01.

White BG		Distilled Water		Coffee		Curry + Oil				
Group	Description	t1-t2	t1-t3	t1-t4	t1-t2	t1-t3	t1-t4	t1-t2	t1-t3	t1-t4
**G_1**	**MeaWhite**	1.67 ^A^ (1.05)	2.44 ^A^ (1.87)	2.97 ^A^ (2.00)	1.84 ^B^ (0.92)	1.97 ^A^ (1.89)	2.89 ^A^ (3.18)	2.74 ^A^ (1.55)	2.64 ^A^ (1.12)	2.66 ^A^ (1.31)
**G_2**	**Iwhite**	1.21 ^A^ (0.79)	1.97 ^A^ (1.9)	2.88 ^A^ (3.33)	3.26 ^B^ (2.42)	3.18 ^A^ (2.2)	3.56 ^A^ (2.61)	2.81 ^A^ (0.93)	3.34 ^A^ (1.22)	3.4 ^A^ (0.69)
**G_3**	**PAP pure**	1.4 ^A^ (0.50)	1.34 ^A^ (0.61)	1.78 ^A^ (0.58)	2.52 ^B^ (1.55)	2.34 ^A^ (1.2)	2.13 ^A^ (1.14)	2.33 ^A^ (0.8)	2.24 ^A^ (0.62)	2.13 ^A^ (0.6)
**G_4**	**Opalescence PF**	2.56 ^A^ (1.11)	2.55 ^A^ (1.59)	3.26 ^A^ (1.53)	2.47 ^B^ (1.28)	2.15 ^A^ (1.32)	3.51 ^A^ (1.95)	2.93 ^A^ (0.75)	2.63 ^A^ (0.95)	3.45 ^A^ (1.02)
**G_5**	**EXP1**	1.36 ^A^ (0.73)	2.01 ^A^ (1.12)	3.04 ^A^ (0.88)	4.24 ^A^ (1.64)	3.02 ^A^ (1.07)	3.69 ^A^ (1.68)	3.3 ^A^ (1.66)	3.41 ^A^ (0.89)	5.07 ^A/B^ (1.71)
**G_6**	**Hismileteeth**	1.32 ^A^ (0.7)	1.35 ^A^ (0.75)	1.90 ^A^ (0.55)	1.64 ^B^ (0.95)	2.1 ^A^ (0.82)	2.13 ^A^ (0.94)	2.76 ^A^ (1.7)	3.00 ^A^ (1.03)	3.67 ^A^ (1.73)
**G_7**	**Placebo**	1.74 ^A^ (1.05)	1.42 ^A^ (0.51)	1.56 ^A^ (0.64)	3.54 ^B^ (5.87)	1.69 ^A^ (0.86)	1.54 ^A^ (0.78)	2.07 ^A^ (0.85)	2.63 ^A^ (0.83)	2.53 ^A^ (1.01)
**G_8**	**oZoral gel**	1.93 ^A^ (1.07)	1.42 ^A^ (0.77)	1.04 ^A^ (0.7)	2.89 ^B^ (2.38)	2.83 ^A^ (2.45)	3.05 ^A^ (2.23)	3.05 ^A^ (1.05)	3.53 ^A^ (1.45)	3.62 ^A^ (1.04)
**White BG**		**Red Wine**		**Tea**		**Overall**				
**Group**	**Description**	**t1-t2**	**t1-t3**	**t1-t4**	**t1-t2**	**t1-t3**	**t1-t4**	**t1-t2**	**t1-t3**	**t1-t4**
**G_1**	**MeaWhite**	5.43 ^B^ (6.79)	4.67 ^B^ (2.29)	5.49 ^C^ (3.15)	3.7 ^B^ (3.51)	2.89 ^C^ (2.77)	2.59 ^C^ (2.82)	3.08 ^B^ (3.69)	2.92 ^C^ (2.2)	3.32 ^C^ (2.74)
**G_2**	**Iwhite**	5.27 ^B^ (2.63)	5.02 ^B^ (3.02)	6.45 ^B^ (2.35)	3.24 ^B^ (4.08)	2.14 ^C^ (1.08)	2.42 ^C^ (1.6)	3.16 ^B^ (2.73)	3.13 ^B^ (2.23)	3.74 ^C^ (2.64)
**G_3**	**PAP pure**	3.83 ^C^ (2.34)	4.57 ^B^ (2.59)	5.12 ^C^ (2.56)	2.08 ^C^ (0.8)	1.94 ^C^ (0.97)	2.21 ^C^ (0.9)	2.43 ^B^ (1.55)	2.49 ^C^ (1.75)	2.67 ^D^ (1.81)
**G_4**	**Opalescence PF**	7.81 ^A^ (3.77)	9.43 ^A^ (4.6)	11.2 ^A^ (4.39)	8.37 ^A^ (6.69)	8.54 ^A^ (7.08)	10.17 ^A^ (6.51)	4.83 ^A^ (4.35)	5.06 ^A^ (4.98)	6.32 ^A^ (5.09)
**G_5**	**EXP1**	4.07 ^C^ (2.26)	3.68 ^B^ (2.46)	5.86 ^C^ (2.24)	3.02 ^B^ (1.41)	2.89 ^C^ (0.97)	4.27 ^B^ (1.31)	3.2 ^B^ (1.87)	3.00 ^B^ (1.49)	4.38 ^B^ (1.86)
**G_6**	**Hismileteeth**	6.44 ^A^ (2.97)	7.58 ^A^ (2.67)	7.7 ^B^ (2.45)	7.99 ^A^ (8.27)	5.28 ^B^ (4.61)	5.31 ^B^ (3.69)	4.03 ^A^ (4.75)	3.86 ^B^ (3.32)	4.14 ^B^ (3.03)
**G_7**	**Placebo**	3.53 ^C^ (1.99)	3.75 ^B^ (3.03)	2.97 ^D^ (1.94)	1.82 ^C^ (0.74)	1.91 ^C^ (1.13)	2.09 ^C^ (1.08)	2.54 ^B^ (2.88)	2.28 ^C^ (1.73)	2.14 ^D^ (1.27)
**G_8**	**oZoral gel**	4.28 ^C^ (3.44)	2.89 ^B^ (1.27)	2.86 ^D^ (1.63)	1.48 ^C^ (1.45)	1.19 ^C^ (1.63)	2.56 ^C^ (2.31)	2.73 ^B^ (2.25)	2.37 ^C^ (1.8)	2.62 ^D^ (1.86)

**Table 5:** Average CIEDE00 color changes over time and standard deviations (in parentheses) per group analyzed over a black background and corresponding grouping (A, best; D, worst). Results with the same capital letter are not significantly different according to Fisher’s LSD test; *p* value < 0.01.

Black BG		Distilled Water			Coffee			Curry + Oil		
Group	Description	t1-t2	t1-t3	t1-t4	t1-t2	t1-t3	t1-t4	t1-t2	t1-t3	t1-t4
**G_1**	**MeaWhite**	2.93 ^A^ (2.33)	2.87 ^A^ (2.57)	3.11 ^A^ (2.34)	1.72 ^A^ (1.19)	1.38 ^B^ (1.05)	3.71 ^A^ (4.26)	3.84 ^A^ (2.4)	3.25 ^A^ (1.55)	3.8 ^B^ (1.91)
**G_2**	**Iwhite**	2.31 ^A^ (2.29)	2.97 ^A^ (2.55)	3.56 ^A^ (3.26)	3.13 ^A^ (1.25)	4.59 ^A^ (1.83)	4 ^A^ (2.81)	2.92 ^A^ (1.69)	3.66 ^A^ (1.8)	3.67 ^B^ (1.05)
**G_3**	**PAP pure**	2.33 ^A^ (1.91)	2.79 ^A^ (2.12)	2.3 ^A^ (1.94)	2.51 ^A^ (1.62)	2.29 ^B^ (1.76)	2.5 ^A^ (1.61)	2.27 ^A^ (1.14)	2.4 ^A^ (1.4)	2.36 ^B^ (1.18)
**G_4**	**Opalescence PF**	4.47 ^A^ (4.27)	4.27 ^A^ (4.44)	4.83 ^A^ (4.41)	3.84 ^A^ (3.37)	3.16 ^A/B^ (2.11)	3.4 ^A^ (1.61)	3.1 ^A^ (1.64)	3.16 ^A^ (1.59)	3.57 ^B^ (1.79)
**G_5**	**EXP1**	2.03 ^A^ (1.09)	2.62 ^A^ (1.89)	3.65 ^A^ (1.24)	3.06 ^A^ (1.21)	2.25 ^B^ (0.94)	3.05 ^A^ (1.64)	3.89 ^A^ (1.58)	3.27 ^A^ (0.85)	6.02 ^A^ (1.91)
**G_6**	**Hismileteeth**	1.52 ^A^ (1.01)	1.29 ^A^ (0.59)	2.08 ^A^ (1.29)	1.93 ^A^ (1.47)	2.17 ^B^ (1.29)	2.11 ^A^ (1.37)	2.81 ^A^ (1.34)	3.19 ^A^ (1.00)	4.17 ^B^ (1.27)
**G_7**	**Placebo**	2.88 ^A^ (1.63)	1.79 ^A^ (1.57)	1.91 ^A^ (1.71)	2.71 ^A^ (1.81)	1.4 ^B^ (0.88)	1.73 ^A^ (1.05)	2.85 ^A^ (1.27)	3.19 ^A^ (1.39)	3.13 ^B^ (1.49)
**G_8**	**oZoral gel**	2.14 ^A^ (1.11)	1.18 ^A^ (0.83)	1.25 ^A^ (0.94)	2.00 ^A^ (1.5)	2.4 ^B^ (1.44)	2.43 ^A^ (1.08)	4.69 ^A^ (1.74)	5.04 ^A^ (2.39)	5.86 ^A^ (1.68)
**Black BG**		**Red Wine**			**Tea**			**Overall**		
**Group**	**Description**	**t1-t2**	**t1-t3**	**t1-t4**	**t1-t2**	**t1-t3**	**t1-t4**	**t1-t2**	**t1-t3**	**t1-t4**
**G_1**	**MeaWhite**	4.77 ^A^ (2.45)	5.55 ^B^ (2.73)	6.28 ^B^ (2.80)	3.92 ^C^ (3.52)	4.53 ^C^ (3.46)	4.37 ^B^ (3.26)	3.44 ^B^ (2.62)	3.51 ^B^ (2.75)	4.25 ^B^ (3.12)
**G_2**	**Iwhite**	4.34 ^A^ (2.04)	5.19 ^B^ (2.51)	5.83 ^B^ (3.10)	3.77 ^C^ (2.92)	3.77 ^C^ (1.84)	4.06 ^B^ (2.13)	3.29 ^B^ (2.16)	4.04 ^B^ (2.20)	4.23 ^B^ (2.64)
**G_3**	**PAP pure**	6.27 ^A^ (3.22)	6.09 ^B^ (3.59)	5.92 ^B^ (4.02)	2.71 ^C^ (2.73)	2.81 ^C^ (2.86)	2.98 ^C^ (2.86)	3.22 ^B^ (2.67)	3.28 ^B^ (2.79)	3.21 ^C^ (2.81)
**G_4**	**Opalescence PF**	6.83 ^A^ (2.57)	8.16 ^A^ (2.94)	9.39 ^A^ (2.59)	6.05 ^B^ (4.11)	6.14 ^B^ (3.6)	7.73 ^A^ (5.1)	4.86 ^A^ (3.51)	4.98 ^A^ (3.57)	5.78 ^A^ (4.07)
**G_5**	**EXP1**	4.78 ^A^ (2.17)	3.77 ^C^ (2.56)	5.28 ^B^ (2.44)	2.72 ^C^ (1.64)	3.10 ^C^ (1.16)	4.47 ^B^ (1.46)	3.30 ^B^ (1.81)	3.00 ^B/C^ (1.65)	4.49 ^B^ (2.04)
**G_6**	**Hismileteeth**	5.40 ^A^ (2.64)	6.34 ^B^ (2.52)	5.43 ^B^ (2.77)	9.97 ^A^ (10.33)	9.17 ^A^ (9.82)	7.50 ^A^ (6.77)	4.33 ^A^ (5.66)	4.43 ^A^ (5.33)	4.26 ^B^ (3.91)
**G_7**	**Placebo**	6.37 ^A^ (3.45)	3.13 ^C^ (1.80)	3.03 ^C^ (1.63)	1.59 ^D^ (0.76)	1.62 ^D^ (0.99)	1.77 ^C^ (1.2)	3.28 ^B^ (2.53)	2.23 ^C^ (1.53)	2.31 ^C^ (1.53)
**G_8**	**oZoral gel**	2.64 ^B^ (1.34)	2.65 ^C^ (0.92)	3.68 ^C^ (1.51)	1.43 ^D^ (0.82)	2.02 ^D^ (1.58)	2.58 ^C^ (2.53)	2.58 ^B^ (1.72)	2.66 ^C^ (1.98)	3.16 ^C^ (2.23)

**Table 6:** Mean and Standard Deviations of CIEDE00 color differences over time according to different staining liquids analyzed over a white background. ^ABCD^ rankings are from the highest (^A^) to the lowest staining results using Fisher’s LSD post hoc test at a *p*-value < 0.01.

	White Background					
	Mean t1-t2	SD t1-t2	Mean t1-t3	SD t1-t3	Mean t1-t4	SD t1-t4
**Distilled water**	1.65 ^D^	0.96	1.81 ^D^	1.30	2.30 ^C^	1.70
**Coffee**	2.80 ^C^	2.64	2.41 ^C^	1.61	2.81 ^C^	2.05
**Curry + oil**	2.75 ^C^	1.23	2.93 ^C^	1.09	3.31 ^B^	1.44
**Red Wine**	5.08 ^A^	3.71	5.20 ^A^	3.46	5.96 ^A^	3.63
**Tea**	3.96 ^B^	4.85	3.35 ^B^	3.89	3.95 ^B^	3.93
**Total value**	**3.25**	**3.26**	**3.14**	**2.79**	**3.67**	**3.02**

**Table 7:** Mean and Standard Deviations of CIEDE00 color differences over time according to different staining liquids analyzed over a black background. ^ABC^ rankings are from the highest (^A^) to the lowest staining results using Fisher’s LSD post hoc test at a *p*-value < 0.01.

	Black Background					
	Mean t1-t2	SD t1-t2	Mean t1-t3	SD t1-t3	Mean t1-t4	SD t1-t4
**Distilled water**	2.58 ^C^	2.27	2.47 ^C^	2.46	2.84 ^C^	2.56
**Coffee**	2.61 ^C^	1.86	2.46 ^C^	1.72	2.87 ^C^	2.23
**Curry + oil**	3.30 ^C^	1.74	3.40 ^B^	1.66	4.07 ^B^	1.92
**Red Wine**	5.17 ^A^	2.78	5.11 ^A^	3.01	5.61 ^A^	3.16
**Tea**	4.02 ^B^	5.01	4.14 ^B^	4.63	4.43 ^B^	4.04
**Total value**	**3.54**	**3.13**	**3.52**	**3.07**	**3.96**	**3.05**

**Table 8:** Mean and Standard Deviations of CIEDE00 color differences (t0-t1) according to different staining liquids analyzed over a white and a black background. ^ABCDE^ rankings are from the highest (^A^) to the lowest staining results using Fisher’s LSD post hoc test at a *p*-value < 0.01.

	White Background		Black Background	
	Mean t0-t1	SD	Mean t0-t1	SD
**Distilled water**	1.85 ^E^	1.34	2.42 ^E^	2.64
**Coffee**	19.05 ^B^	8.74	18.88 ^B^	9.09
**Curry + oil**	9.60 ^C^	3.44	10.48 ^C^	4.32
**Red Wine**	21.67 ^A^	6.39	21.30 ^A^	6.45
**Tea**	7.60 ^D^	5.24	7.25 ^D^	4.42

**Table 9:** L*a*b* change over time for every bleaching agent over a black and a white background.

**L**	**BLACK**					
		**t0**	**t1**	**t2**	**t3**	**t4**
	**G1**	**77.36**	**64.51**	**66.05**	**66.67**	**67.25**
	**G2**	**77.70**	**66.68**	**69.29**	**70.56**	**70.87**
	**G3**	**77.93**	**67.44**	**69.05**	**68.97**	**69.02**
	**G4**	**76.85**	**66.16**	**70.16**	**70.45**	**71.85**
	**G5**	**77.96**	**67.09**	**68.18**	**68.85**	**70.55**
	**G6**	**77.55**	**66.47**	**64.02**	**63.90**	**64.96**
	**G7**	**77.79**	**66.73**	**67.91**	**67.72**	**67.65**
	**G8**	**76.97**	**55.76**	**56.10**	**56.41**	**57.05**
	**WHITE**					
		**t0**	**t1**	**t2**	**t3**	**t4**
	**G1**	**78.42**	**65.81**	**67.04**	**67.55**	**68.01**
	**G2**	**79.14**	**68.19**	**70.52**	**71.06**	**71.78**
	**G3**	**80.58**	**69.08**	**70.47**	**70.81**	**70.41**
	**G4**	**78.14**	**67.12**	**71.12**	**71.55**	**73.22**
	**G5**	**79.47**	**68.07**	**69.69**	**69.99**	**71.62**
	**G6**	**79.18**	**67.80**	**65.45**	**65.63**	**65.84**
	**G7**	**79.36**	**67.88**	**68.36**	**68.60**	**69.19**
	**G8**	**80.01**	**58.31**	**58.16**	**58.93**	**58.96**
**a**	**BLACK**					
		**t0**	**t1**	**t2**	**t3**	**t4**
	**G1**	2.49	4.57	4.18	4.24	4.17
	**G2**	2.06	3.71	3.49	3.33	2.68
	**G3**	1.36	3.11	2.55	2.51	2.64
	**G4**	2.54	4.20	2.64	2.49	2.25
	**G5**	1.07	3.20	2.96	2.53	2.20
	**G6**	1.46	3.85	4.34	4.18	4.14
	**G7**	1.58	3.76	2.99	3.33	3.27
	**G8**	0.65	6.29	5.40	5.46	5.17
	**WHITE**					
		**t0**	**t1**	**t2**	**t3**	**t4**
	**G1**	3.10	5.19	5.55	4.73	4.53
	**G2**	2.55	4.22	4.08	4.02	3.33
	**G3**	2.01	3.95	3.34	3.23	3.34
	**G4**	3.15	4.63	3.21	3.12	2.76
	**G5**	1.70	3.92	3.44	3.00	2.76
	**G6**	2.03	4.27	4.94	4.73	4.84
	**G7**	2.21	4.08	3.78	3.83	3.67
	**G8**	1.41	6.67	5.65	5.92	5.61
b	**BLACK**					
		**t0**	**t1**	**t2**	**t3**	**t4**
	**G1**	16.21	17.02	17.49	17.50	17.07
	**G2**	14.99	18.14	17.44	16.88	15.96
	**G3**	13.50	17.00	15.74	15.94	16.30
	**G4**	16.29	18.54	16.38	16.46	15.71
	**G5**	15.56	18.20	17.54	16.81	16.13
	**G6**	15.20	18.47	17.91	17.28	16.59
	**G7**	14.04	15.71	14.06	15.31	14.99
	**G8**	15.02	19.19	19.15	18.88	18.39
	**WHITE**					
		**t0**	**t1**	**t2**	**t3**	**t4**
	**G1**	17.37	18.65	18.31	18.47	18.15
	**G2**	16.33	19.42	18.96	18.44	17.23
	**G3**	15.24	18.47	17.37	17.67	17.90
	**G4**	17.69	19.91	17.87	17.58	16.60
	**G5**	17.10	19.62	18.68	17.94	17.33
	**G6**	16.63	19.67	19.24	18.51	18.09
	**G7**	15.58	16.23	15.49	16.14	15.79
	**G8**	17.39	20.90	20.07	20.16	19.56

## Data Availability

All data are available in the manuscript.
